# Improving community development by linking agriculture, nutrition and education: design of a randomised trial of “home-grown” school feeding in Mali

**DOI:** 10.1186/1745-6215-14-55

**Published:** 2013-02-21

**Authors:** Edoardo Masset, Aulo Gelli

**Affiliations:** 1 Institute of Development Studies, University of Sussex, Brighton BN1 9RE, UK

**Keywords:** School feeding, Impact evaluation, Education, Nutrition, Agriculture

## Abstract

**Background:**

Providing food through schools has well documented effects in terms of the education, health and nutrition of school children. However, there is limited evidence in terms of the benefits of providing a reliable market for small-holder farmers through “home-grown” school feeding approaches. This study aims to evaluate the impact of school feeding programmes sourced from small-holder farmers on small-holder food security, as well as on school children’s education, health and nutrition in Mali. In addition, this study will examine the links between social accountability and programme performance.

**Design:**

This is a field experiment planned around the scale-up of the national school feeding programme, involving 116 primary schools in 58 communities in food insecure areas of Mali. The randomly assigned interventions are: 1) a school feeding programme group, including schools and villages where the standard government programme is implemented; 2) a “home-grown” school feeding and social accountability group, including schools and villages where the programme is implemented in addition to training of community based organisations and local government; and 3) the control group, including schools and household from villages where the intervention will be delayed by at least two years, preferably without informing schools and households. Primary outcomes include small-holder farmer income, school participation and learning, and community involvement in the programme. Other outcomes include nutritional status and diet-diversity. The evaluation will follow a mixed method approach, including household, school and village level surveys as well as focus group discussions with small-holder farmers, school children, parents and community members. The impact evaluation will be incorporated within the national monitoring and evaluation (M&E) system strengthening activities that are currently underway in Mali. Baselines surveys are planned for 2012. A monthly process monitoring visits, spot checks and quarterly reporting will be undertaken as part of the regular programme monitoring activities. Evaluation surveys are planned for 2014.

**Discussion:**

National governments in sub-Saharan Africa have demonstrated strong leadership in the response to the recent food and financial crises by scaling-up school feeding programmes. “Home-grown” school feeding programmes have the potential to link the increased demand for school feeding goods and services to community-based stakeholders, including small-holder farmers and women’s groups. Alongside assessing the more traditional benefits to school children, this evaluation will be the first to examine the impact of linking school food service provision to small-holder farmer income, as well as the link between community level engagement and programme performance.

**Trial registration:**

ISRCTN76705891

## Background

Today, every country for which we have information is seeking to provide food, in some way and at some scale, to its schoolchildren. However, where the need is greatest, in terms of hunger, poverty and poor social indicators, the programmes tend to be the smallest. Past experience shows that countries do not seek to exit from providing food to their schoolchildren, but rather to transition from externally supported projects to nationally-owned programmes [1]. Countries that have made a successful transition have often explored linking school feeding programmes to agriculture development – an approach also known as “Home Grown School Feeding” (HGSF) [2].

Strategic leadership from the New Partnership for Africa’s Development (NEPAD) guided Governments in Sub-Saharan Africa to include HGSF as a key intervention within the food security pillar of the Comprehensive Africa Agriculture Development Programme (CAADP) framework. Several countries, including Cote d’Ivoire, Ghana, Kenya, Mali and Nigeria are already implementing national programmes. From this perspective, HGSF provides an integrated framework with multiple impacts across agriculture, health, nutrition and education [3]. Since early 2008, the World Bank Group, World Food Programme (WFP) and the Partnership for Child Development (PCD) have been working together to help governments develop and implement cost effective, sustainable, national school feeding programs.

How best can the potential of school feeding be maximised to support multi-sectoral integrated frameworks linking agriculture, health, nutrition and education? Can HGSF be a win-win for agriculture, education and health? There is a need to answer these questions operationally and build the evidence base to help policy makers manage the trade-offs across the multiple school feeding objectives [4]. This paper develops the design of a field experiment of the HGSF programme in Mali, with baselines planned for 2012.

### Mali country context

Mali is, according to the Food and Agricultural Organization of the United Nations (FAO) definition, a Low-Income Food Deficit Country (LIFDC) with a population of 14 million people, over half of whom are under 15 years of age. According to the United Nations Development Programme (UNDP), Mali is ranked 178^th^ in the Human Development Index table, with an average life expectancy at birth of 48 years, adult literacy rate of 26 percent and a gross domestic product (GDP) per capita (PPP) of $1,083 USD. At a country level, Mali has seen remarkable progress in terms of access to school (net enrolment ratios increased from 20% in 1990 to 66% in 2007) but the levels of enrolment are still well below the average for Sub-Saharan Africa and completion rates are very poor^1^. A large proportion of children, girls in particular, are excluded from the schooling system. There are also large disparities within Mali; in the regions of Koulikoro and Mopti, for example, girls’ enrolment was estimated at 44% in 2005 [5].

Agricultural productivity in Mali is among the lowest in the world [6]. In Mali, the majority of farmers are involved in the production of food crops, with the main cereals being millet and sorghum. Production is carried out using a low level of technology: fertiliser use is minimal and access to credit is limited. Crop yields are not only low but also highly variable due to the fact that most farmers depend on rain-fed farming while rainfall fluctuates considerably from year to year and season to season [6].

According to the 2005 WFP food security and vulnerability analysis, an estimated 4 million people, or 40% of the population live in food insecurity or are highly vulnerable to food insecurity. According to this assessment, the regions most at risk are Kayes, Koulikoro, Mopti, Tombouctou, Gao and Kidal. The food security assessment also showed that food access is a primary constraint: food is available at markets when harvests are good but populations face constraints in food access and utilisation.

Thirty-eight percent of children under five years of age are chronically malnourished or stunted in their growth (low height for age), 15% are acutely malnourished or wasted (low weight for height), and 27% are underweight (low weight for age), which is a composite measure of stunting and wasting^2^. The majority (81%) of children 6 to 59 months of age are anaemic^3^, caused mainly by iron deficiency, malaria and helminth infections. Anaemia prevalence in school-age children is lower but still unacceptably high with 56% of school children affected [7]. Thirty-seven percent of five year olds, when they will soon be entering primary school, are stunted in their growth and eight percent are wasted.

## Design of the intervention

The interventions involve school feeding implemented by the government of Mali. The national programme was launched in 2009 and currently targets 651 schools located mainly in the 166 most vulnerable communes (official data report that currently 10% of the 9,400 primary schools in the country have a school canteen run by the state or by partners, including WFP and Catholic Relief Services (CRS)). The targets of the school feeding intervention are public primary schools of rural villages and children attending these schools. Compulsory basic education in Mali is composed of six years of primary and three years of lower secondary. The schooling age for primary is 7 to 12 and it is 13 to 15 for lower secondary. Considering the high repetition rate (15 to 20%), early and delayed entrants, and uncertainties and misreporting of age, the target group of intervention consists of children aged 5 to 17 and their families.

### The national school feeding programme in Mali

In line with the National Decentralisation Policy and the 2009 National School Feeding Policy, programme implementation is decentralised to the community level, involving the communes and School Management Committees (CGS). The central government allocated nearly 5 million USD (2.6 billion CFA) in 2010 and 5.8 million USD in 2011 (3.1 billion CFA) from the national budget for food, cooking equipment and infrastructure rehabilitation and construction [8]. Funds are channelled directly through the Ministry of Finance to its Regional Offices which in turn send funds to the communes. Food commodities are procured from local markets by the communes or by the CGS. To date, there is no fixed food basket nor fixed ration specification. The budget allocated for food procurement is based on student enrolment figures obtained by the Ministry of Education and price estimates for staples at the beginning of the school year. The budget covers staples, including cereals and pulses and oil. Fresh vegetables to complement the school feeding ration are contributed by parents and the community through cash or in-kind contributions. Cooks are generally organised on a voluntary basis (also considered as the contribution to the program from the community) through the CGS.

**Figure 1:**
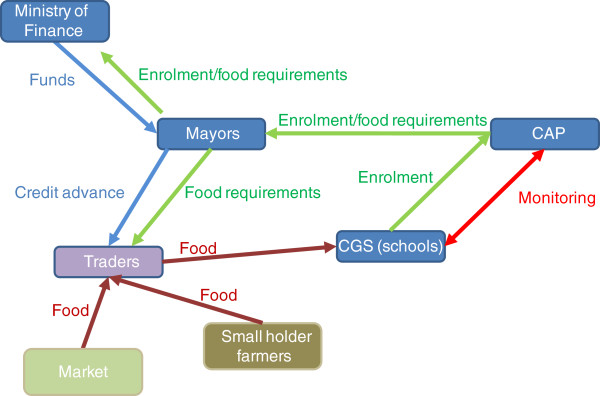
Stylised food procurement process in the national programme.

Food provision consists of a school lunch meal served at noon (school is from 8 am to 12 am and then again from 3 pm to 5 pm). The food mainly consists of staples (rice, millet or sorghum) enriched with condiments, vegetables, vitamins and minerals depending on the source of provision. Figure 1 captures the key activities in the food procurement process currently in use in the national programme. Enrolment figures are collected by the Centres d’ Animation Pedagogicue (CAP), the equivalent of district education offices, through the CGS and passed on to the mayors to estimate the food requirements for school feeding. Mayors receive budgets from the Ministry of Finance on the basis of the enrolment/food requirements, then issue tenders, on the basis of a credit advance, to certified service providers (traders) to procure the food. The service providers (traders) then purchase the food from the market or from small-holders, and deliver it to the relevant schools.

Mayors are public officials representing the democratically elected local authority within each commune. The office of the Mayor is responsible for the food procurement in all the relevant schools in the commune.

The purchase of food by mayors presents a number of problems [9]:

•Food is mostly purchased from traders rather than from small-holder farmers

•Food purchased by mayors is often unrelated to food habits of the beneficiary communities; it does not follow any specific nutritional advice, and its quality is uncertain

•Food is delivered to communities with delays and CGS are uncertain about deliveries

•Food can be easily in excess or deficit of needs because it is purchased based on rough estimates of enrolment (with low enrolment rates the margin of error can be large and the programme has the effect of increasing enrolment which is not accounted for in the allocation)^4^

### The HGSF pilot

**Figure 2:**
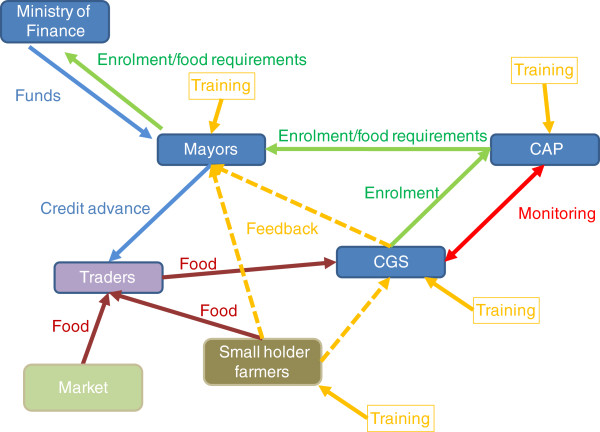
Stylised food procurement process in the HGSF+ programme.

An innovative capacity-building component will be integrated alongside the national school feeding programme and will constitute one of the treatment arms of the experiment (see Figure 2). The HGSF pilot intends to promote purchases from small farmers through training, monitoring and communication activities among the actors involved. Mayors and CAPs will be instructed, and encouraged, to involve small farmers in the transactions. Currently, mayors purchase food from one or more traders following government guidelines regarding contracts and bidding processes. Under the project, and in coordination with the Ministry of Agriculture, mayors and traders will be requested to enforce the purchase of food from local producers at least at a minimum percentage. On the other hand, the CGSs will be assigned the task of identifying local suppliers, possibly at the village level, and of establishing contacts between these suppliers and the traders.

A working group composed of in-country stakeholders, including the Dutch Cooperation Agency (SNV), Catholic Relief Services (CRS), and WFP is currently working on the details of the package of “software” interventions to improve overall programme performance. This package will include:

•Training of mayors and CAPS. At the beginning of each school year a number of training events will be organised for Commune-level stakeholders, including mayors and CAP. The purpose of the training events is to improve service delivery on several accounts:

◦ The establishment of a monitoring system of programme activities

◦ The identification of formulas for the allocation of food to schools (currently the allocation is based on rough enrolment estimates, but enrolment is endogenous as it is driven by food provision, hence a flexible system of funds and food allocation based on ‘predicted’ enrolment needs to be put in place.)

◦ The illustration of methodologies for purchasing from smallholder farmers rather than from traders (formulation of bids, contracts, contingency plans and so on)

◦ Nutrition education and the importance of adequate nutrition for school children and smallholder farmers, for example, how to use the foods they grow or purchase to improve the nutritional status of all family members, including diet diversification, availability of staple and nutrient rich foods by season, food storage and safety, food processing and preparation.

•Training of CGS. Training events for CGS at the village level will be held periodically, on a monthly or quarterly basis, in every intervention village:

◦ Members of CGS will be given the instruments to efficiently implement the programme including basic accountancy skills, information on their rights and entitlements, and information on methods to raise complaints

◦ Participatory monitoring events will be periodically held at the village level. +Members of CGS, parent teacher associations (PTAs), and parents will be involved in participatory events where the programme performance will be monitored in close detail, suggestions for improvement will be proposed and complaints brought to the attention of project staff and CAPs.

◦ Nutrition education and the importance of adequate nutrition for school children and smallholder farmers, for example, how to use the foods they grow or purchase to improve the nutritional status of all family members, including diet diversification, availability of staple and nutrient rich foods by season, food storage and safety, food processing and preparation.

## Programme theory of the intervention

School feeding interventions linked to small-holder agriculture can have multiple goals in the following areas:

•Food security: supporting incomes of recipient households (those consuming food) and farmer households (those providing the food)

•Education: increasing school enrolment, attendance and reducing drop-out, and improving cognition and learning achievement

•Health: improving nutritional status of school age children

The impact of the intervention in each of the above areas occurs through a number of complex pathways. This section describes the pathways through which the programme is expected to operate. Though the evaluation of all potential effects of the intervention is beyond the scope of this study, a subset of outcome indicators for the evaluation will be selected based on: a) a knowledge gap specific to school feeding in Mali; b) knowledge gaps in the school feeding literature in general; c) feedback from peer reviewers and technical partners; and d) budget constraints.

**Figure 3:**
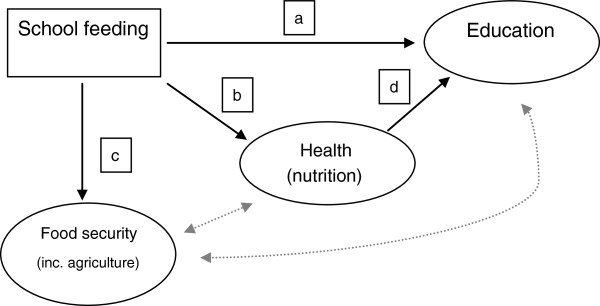
Overall programme theory of school feeding interventions.

Figure 3 illustrates in very broad terms the impact theory of school feeding on food security, education and health. School feeding affects educational outcomes directly by increasing enrolment, attendance and completion (line ‘a’ in the figure). It affects health directly by improving nutritional status (line ‘b’), this in turn has an indirect impact on education, as improving nutritional status has a positive impact on learning outcomes (line ‘d’). The intervention can also affect income directly by increasing households’ food security (line ‘c’). Finally, there are effects running through increased income, health and nutrition, and vice versa, as richer families are investing more in human capital and more educated and healthier adults are more economically productive. However, these latter effects only occur in the long term and certainly not before children have left school; therefore, we will not discuss them in the following design.

It must be emphasised that the ability of the school feeding intervention to deliver the effects depicted in Figure 3 critically depends on the appropriate implementation of the programme. The management and implementation of the intervention involves several actors and scoping visits. A preliminary study undertaken by PCD [9] has shown that in Mali there are several problems of communication, supervision and monitoring among these different stakeholders. Programme success will also depend on the ability of communities to actively engage in the programme and in the strengthening of the public institutions involved. The issues of social accountability, ‘good governance’ and the links between accountability and programme effectiveness are important areas that this impact evaluation will explore in more detail.

### Impact on food security and smallholder agriculture

**Figure 4:**
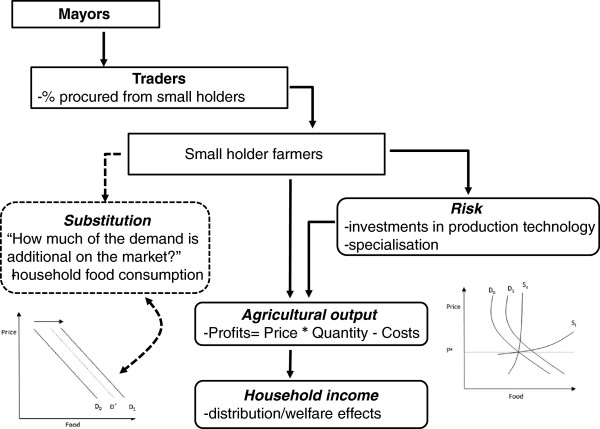
Programme theory of school feeding and smallholder agriculture.

The intervention is designed to stimulate the economy at a community level by purchasing food from small-holder farmers. Food for the school feeding programme is currently purchased from traders by mayors of the Communes of intervention. Traders in turn purchase food from small-holder producers, though these do not need to be residents of the villages targeted by the project. The capacity building or HGSF+ component supported by PCD is intended to confer the community-based organisation more decision power and will increase their ability to purchase from individual farmers or farmers’ associations residing in the project villages. On the small-holder farmer production side the programme can have three main effects that are summarised in Table 1 and schematically in Figure 4, including output effects, distributional effects and stabilisation effects. In addition to these effects, the programme can also have some wider effects on the local economy by generating employment.

**Table 1:** Programme impact on smallholder farmers

** Effects**	**Impact on smallholder farmers**	** Spill-over effects**
Output effects	Increase in farm profits	Increase in consumer prices
Distributional effects	Increase in farm profits	Decrease in large farmers’ profits
Stabilisation effects	Risk reduction	none

**Figure 5:**
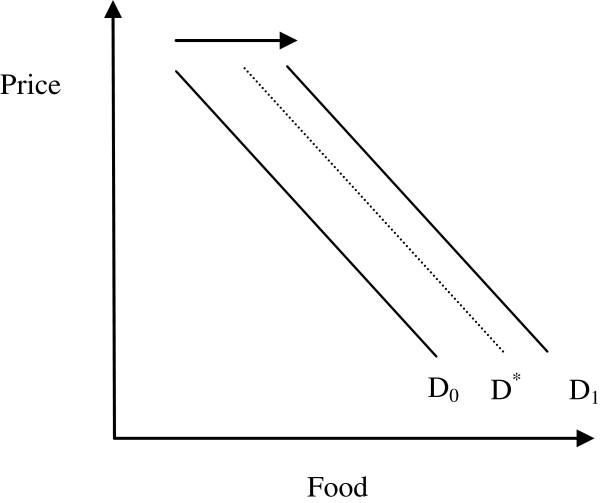
Potential impact of HGSF on commune food demand.

The programme introduces additional demand on the market. In Mali, the school feeding programme purchases food for schools at the Commune level, which includes several villages. Most Communes have food markets. Mayors purchase from traders in the commune. Traders in turn purchase food from, among other sources, small-holder farmers. The effect of the food purchases is a shift of the demand curve for food (say rice) to the right (movement from D_0_ to D_1_), as illustrated in Figure 5.

The size of the shift depends on the extent of the substitution effects and on the size of the market considered. Substitution occurs when households reduce the domestic consumption of food because children are fed in school. At one extreme, there is full substitution: families do not provide any additional food to children fed in school or any other household member in excess of the school food ration. Food provided in school entirely substitutes for food normally consumed in the home. The result is an increase in household savings and consumption of non-food items. In this case, there is no shift of the demand function (D_0_) and HGSF does not have price or output effects. There is still a distributional effect of the intervention if food is purchased from small-holders rather than large farmers. Profits of large farmers will in this case decrease whilst profits of smallholder farmers increase. Full substitution, however, is unlikely to occur. The largest substitution is likely to occur when households interpret the school food ration as a cash transfer. In this, theoretical, case the income equivalent of the ration is spent following income elasticities. Considering that the areas of intervention are very poor, assuming an income elasticity of food of 0.6 to 0.8 and a food share of 0.8, we understand that only about half of the HGSF transfer would eventually be spent on food and the food demand function shifts to D^*^. However, studies show that rarely do people interpret food transfers as cash transfers and that people tend to attach some preference to the food received and thus consuming food beyond what the income elasticities would suggest. Therefore, the final shift in the demand curve is likely to happen somewhere between the curves D^*^ and D_1_.

The size of the demand shift also depends on the size of the market considered. If we are considering the national rice market, the shift is extremely small and the price and output effects are likely to be negligible [10]. The effect is larger at the commune level if food is procured locally. However, some of the food may be purchased outside the commune and, therefore, the shift of the demand function at the commune level would be smaller than the one depicted in Figure 5.

**Figure 6:**
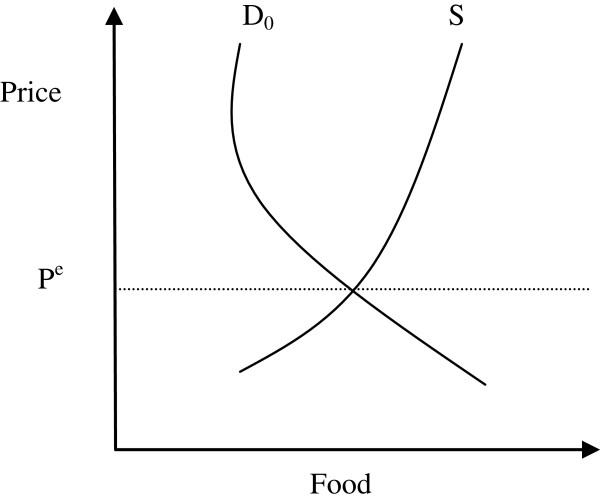
Illustrative view of the commune food demand market and HGSF.

The impact of HGSF on output and prices at the commune level will depend on the slopes of the demand and supply functions (see Figure 6). Welfare effects on producers and consumers can be calculated using changes in consumer and producer surpluses. We do not know the values of the supply and demand elasticities of food items, which need to be estimated econometrically. For convenience, we consider constant elasticities around the equilibrium point (a logarithmic form provides this type of elasticity ln(*q*) = *a* + *bln*(*p*) + *cln*(*y*).

Consumer surplus is the area between the market equilibrium price line (P^e^) and the demand curve (D_0_). Producer surplus is the area between the market equilibrium price line (P^e^) and the supply curve (S). Producers’ and consumers’ surpluses can be calculated provided we know the initial equilibrium price level (P^e^), the quantities of food produced and consumed at this price, and the own price elasticity of food demand (the shape of the demand and supply curves).

**Figure 7:**
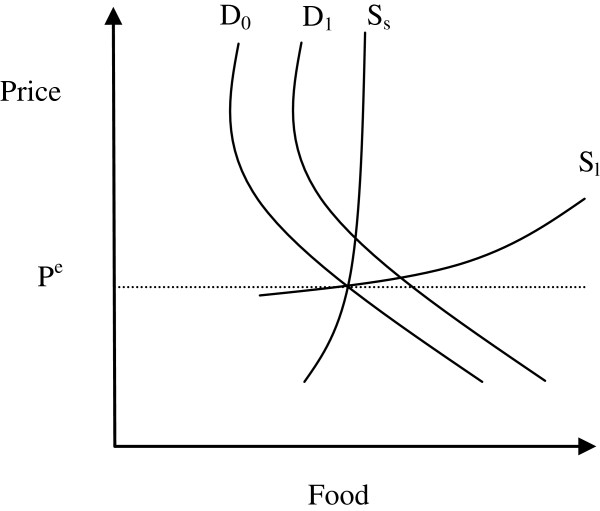
Potential impacts of HGSF with small and large supply elasticities.

Two extreme cases of small and large supply elasticities are shown in Figure 7. The size of supply elasticity will depend on three main factors, such as yield risk, market failures and rigidity of fixed factors. High income risk and missing markets are likely to be present in the communes of intervention, thus reducing the size of supply elasticity. Farmers are not likely to respond promptly to price changes. In addition, while farmers may be able to vary the amount of variable inputs used (labour and fertiliser, for example), they might not be able to change the amount of fixed input in the short run (such as, equipment, land and livestock).

The two supply curves in Figure 7 can be seen as short- and long-term supply curves or elastic and inelastic with respect to risk and market factors. In any case, they illustrate the differential impact of HGSF on prices and output.

•If farmers are not able to provide the additional food demanded by HGSF (small supply elasticity – curve S_s_), then most of the effect of HGSF goes into prices and little impact on output. From a welfare perspective, producer surplus increases (farmers win), while consumer surplus may decrease (consumers may lose).

•If farmers provide any additional food demanded by using current input more intensively and by quickly changing use of fixed inputs (large supply elasticity – curve S_l_), then HGSF would have a large impact on output and a negligible impact on prices. From a welfare perspective producer surplus increases (farmers win) and consumer surplus increases as well (consumers win). For the benefit of both producers and consumers, therefore, a high supply elasticity is needed.

In general, we should expect the supply elasticity to be between the two extremes depicted and, therefore, the programme to have an impact on both prices and output. The impact on prices depends on the level of spatial market integration. In principle, if markets are efficient, prices for the same food items should be the same everywhere after an adjustment for transport costs. However, the literature on market integration suggests that transfer costs may create a wedge between prices at different locations, which allow prices in the two locations to vary in an uncorrelated way within a band [11]. In other words, if transport costs for an isolated commune are very high, food prices may increase up to a point when trade between the two locations takes place and prices are equalised. There is, therefore, a real possibility that food prices increase at the commune level and that these price effects are transmitted to consumers.

Based on this theoretical model, the programme will have a positive impact on farmers’ income via an increase in prices and food quantities produced. The impact on consumers is less obvious. Depending on the size of the increase in prices, some households may have their welfare reduced as a result of the intervention. This observation suggests that the evaluation in addition to assessing impact on farmers’ income should also monitor price levels and model, by simulations, if not observations, their impact on consumers. Note also that the intervention will have other minor positive effects at the village level by creating additional employment (cooks, treasurers and stock keepers) and demand. This suggests that a micro-simulation at the village level should be conducted in order to assess the potential impact through general equilibrium effects.

The programme also has a distributional impact as it shifts demand from large to small farmers. As described in earlier sections, even if households are fully substituting the school meal, the programme generates a demand shift from large to small farmers. While small local farmers see an increase in their income, larger farmers suffer a reduction. This effect can be observed to the extent that the evaluation will be able to collect income data from a large number of farmers, with and without the programme, large and small.

Finally, the programme potentially reduces household risk. The programme can stabilise small farmers’ incomes by offering a stable demand and price. A number of positive effects follow from risk reduction, including an increase in expected utility, a reduction in the use of inefficient mitigating and coping strategies, (such as lower yielding crops and precautionary savings), and an increase in productive investments. This impact can be observed indirectly by observing farmers’ risk-mitigating and coping behaviours. However, it is quite possible that yield risk dominates price risk. In addition, whatever the price effects, these may take a long time before having an impact on farmers’ expectations. The programme’s impact on risk behaviour, therefore, is unlikely to be large.

**Figure 8:**
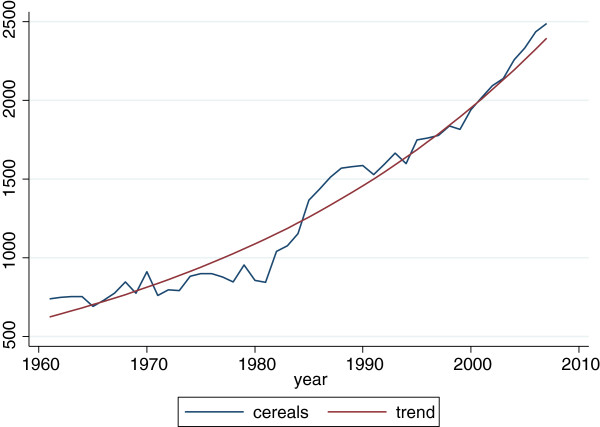
Cereal production in Mali 1960 to 2007; Source: Data from FAO.

**Figure 9:**
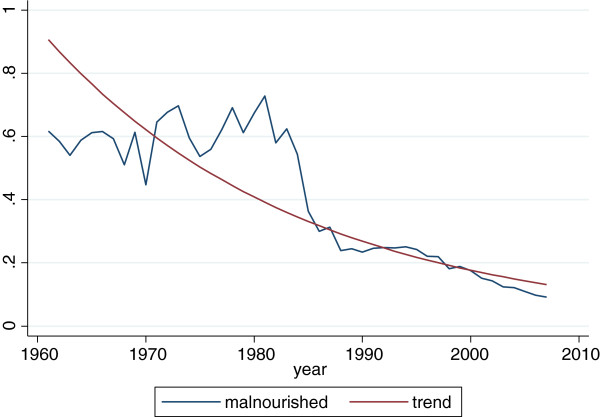
Malnutrition in Mali from 1961 to 2007; Source: Calculated from FAO data.

It is less obvious that a stabilisation of income variability at the aggregate level is needed or is an obstacle to the implementation of the programme. According to an USDA report [6], the success of HGSF initiatives in Mali is potentially compromised by the insufficiency and instability of food production in the aggregate, by the inability of vulnerable regions and areas to produce food in the desired quantity and at the desired time, and by the inability of farmers to respond to the incentives provided by the project. Our analysis of the Food and Agriculture Organisation (FAO) data on aggregate cereal production in Mali over the period 1961 to 2007 suggests that, at current growth rates, by the year 2017 agricultural production in the country will be sufficient to bring malnutrition below 5% of the total population (see Figures 8 and 9). It is also clear that cereal production has not only dramatically increased from the mid-80s but has also become more stable, accompanied with reduction in undernutrition rates during the same period.

The sufficiency and stability of the food supply in the aggregate may hide seasonal stress, regional differences and the presence of chronically poor groups. However, in each village, the pool of farmers from which the project will purchase food is rather small and the ultimate sellers will be traditionally surplus farmers who in the absence of the programme would sell to traders. Two other factors may facilitate a stable supply of food at the community level. First, the involvement of the community-based organisation in the management of the intervention will help the identification of farmers able to provide food at the desired time and in the desired amounts. Second, following a model adopted by the *Purchase for Progress* programme of the WFP, purchases may be organised through contracts with farmers’ associations rather than with individual farmers, thus increasing the likelihood of providing a stable supply.

### Impact on education

**Figure 10:**
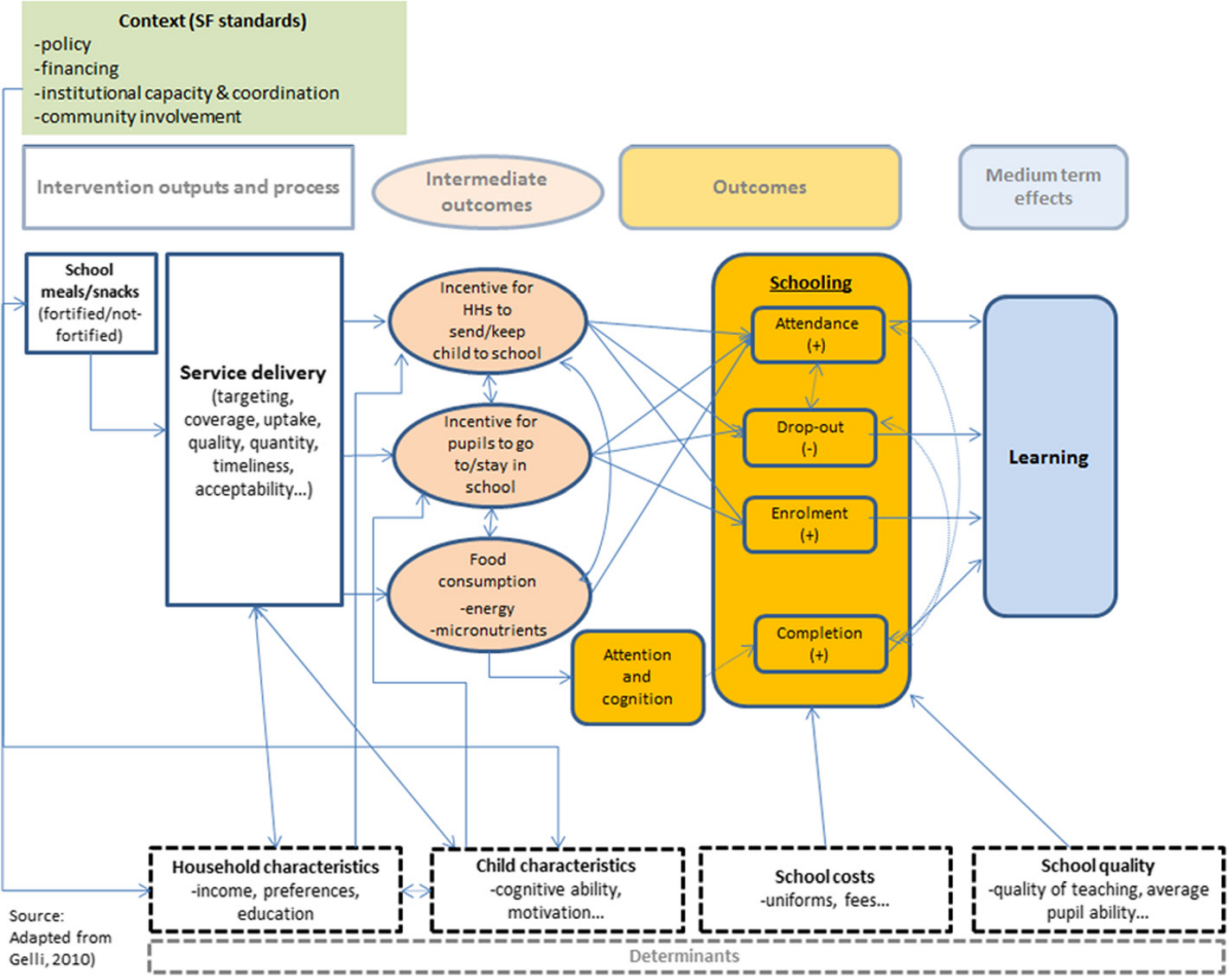
**Programme theory of impact on education.** The main determinants of schooling and learning are child characteristics, schooling costs, households’ characteristics and school quality. Children do not participate in schooling for different reasons; at the household level, it is often a trade-off between the costs and benefits of schooling that determine whether a child will go to school or not.

We formulate hypotheses regarding the impact of school feeding on child schooling and learning starting from an economic model of parental educational choices in developing countries adapted from [12]. Figure 10 below illustrates the determinants of schooling and learning. Schooling produces learning, which in turn has welfare effects. Schooling can be thought of as enrolment, attendance, drop-out or school completion. Learning is the acquiring of basic skills, such as language and mathematics. These skills are valued in the markets and educated children are expected to generate higher incomes and wages. In addition, more educated individuals may conduct healthier lives.

In this model, the main determinants of schooling and learning are child characteristics, schooling costs, households’ characteristics and school quality. Cognitive ability and motivation facilitate learning and encourage families to send children to school. Children do not participate in schooling for various reasons; at the household level, it is often a trade-off between the costs and benefits of schooling that determine whether a child will go to school or not. Costs are not only direct, such as school fees. For example, the opportunity cost of sending a child to school would mean foregoing the benefits of any work that the child could be doing instead of attending school. Often, the opportunity costs follow seasonal patterns, or increase with age, meaning that older children might need stronger incentives than younger children in order to stay in school. The opportunity costs of schooling may also be higher for girls - girls are often kept at home to look after siblings, help with other work, or simply for cultural reasons. These costs have a direct effect on schooling but should not affect learning. Household characteristics such as income and preferences (including attitudes towards education, and time discounting) affect schooling directly, while other characteristics may affect learning directly (for example, more educated parents may improve learning by helping children with their homework). School quality affects learning directly through the quality of the teaching, the teaching environment (supplies and facilities) and schooling, by affecting household schooling decisions.

An initial outcome that drives increased school participation is the incentive to households to send children to school. Generally, this incentive is achieved through an income transfer offsetting the financial and opportunity costs of schooling, and also through an enhancement of the services provided at school. School feeding may also have an incentive effect on pupils actually wanting to go to school to receive food rather than staying at home and missing out. In theory, both of these effects will contribute to shift short-term household decisions towards increased schooling. The specific effect of the incentive will very much depend on the context in which school feeding is operating. Conceptually, the health and nutrition improvements from school feeding can also reinforce the impact on education. Addressing micronutrient deficiencies, in particular iron and iodine, has been shown to have a positive impact on learning (see Section Impact on nutrition), as has the systematic deworming of school-age children in areas of high prevalence of intestinal helminths [13]. The income transfer incentive and the improved health and nutrition status resulting from school feeding service provision would then lead to improved access and learning outcomes.

Learning increases as a result of schooling. In addition, the same factors affecting parents’ decisions also affect learning directly. In this model, school feeding affects schooling and learning in two ways:

•School meals reduce financial and opportunity costs of schooling and, therefore, increase schooling directly, which in turn affects learning positively.

•School meals increase cognitive ability. This in turn increases child learning in school and affects parents’ schooling decisions (learning increases expected income and, therefore, parents’ interest in schooling).

Note that the reduction in school costs can be partially outweighed by additional programme costs. In particular, the programme may require community participation in two ways [1,8,9]. First, communities are sometimes required to provide fire-wood for cooking and other items such as fresh fruit, vegetables and condiments [14]. In addition, they are expected to provide cooks and storekeepers (though participants in these activities often receive compensation in the form of a daily meal). Second, the CGS may collect contributions from parents either in monetary form or in-kind. All these contributions increase the costs of schooling.^5^

There may also be feedbacks from increased schooling and learning to school quality. First, learning in school may increase because the average cognitive ability of pupils has increased (peer effect) or because teachers become more motivated to teach. Second, cost reduction may bring to school children of poorer background thus reducing the average cognitive ability and reducing the overall performance via the same peer effects. Third, schools may become overcrowded because of increased attendance, though the effects of crowded classrooms on learning are still unclear [15].

### Impact on nutrition

**Figure 11:**
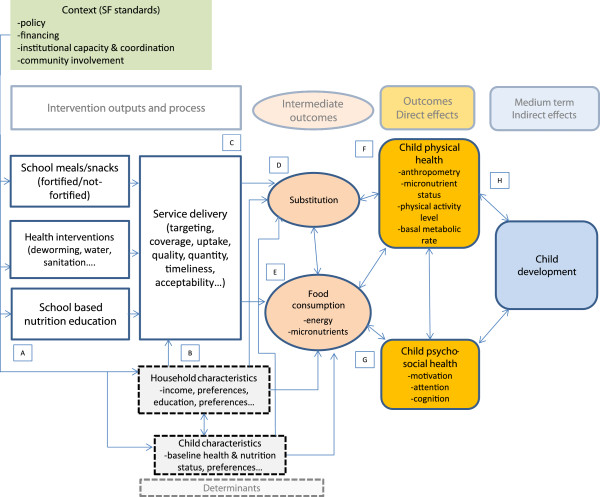
**Programme theory of impact on nutrition.** The nutritional impact is mediated by the extent of food substitution effects within the household, and the use of the energy intake by the child and her siblings. The reduction in malnutrition via diet diversification and the absorption of micronutrients in the body can have direct effects on cognition. Better nourished children may learn better while in class and outside class.

School feeding interventions can potentially have an impact on nutritional status of school children and their younger siblings, as summarised in Figure 11.

a) The nutritional impact is mediated by the extent of food substitution effects within the household, and the use of the energy intake by the child and her siblings.

b) The reduction in malnutrition via diet diversification and the absorption of micronutrients in the body can have direct effects on cognition.

c) Better nourished children may be best positioned to learn while in class and outside class.

These three issues will now be discussed:

a. Substitution effects

Building on [17,18], we provide a simplified programme theory of the nutritional impact of school feeding. The school meal can be shared by children with other household members or can substitute (at least partly) for food normally consumed in the home. This is obvious in the case of take-home-rations, whereby children take home a quantity of food on a regular basis, but also applies to any school feeding programme, because households may in principle use the school meal as a substitute for food normally consumed and spend the monetary equivalent otherwise. Evaluations of fortified biscuit programmes in Bangladesh and Indonesia found that gains in nutritional intake were not limited to the children actually receiving the biscuits at school. The two studies found significant evidence that school children shared the biscuits with their younger sisters or brothers at home. A recent randomised control trial (RCT) in Burkina Faso also found that take-home-ration programmes led to an improvement of the nutritional status of younger siblings in beneficiary households [19]. In Uganda, an RCT also found significant improvements for pre-schooler siblings of children receiving school feeding [20]. This provides emerging evidence of a spill-over effect and a window of opportunity to also affect children during a critical developmental stage when nutritional interventions can have the strongest impact.

Ingested foods contribute to three outcomes, of which physical growth is only one:

••Physical growth. Food can help physical growth in terms of height and weight. It is normally believed that catching-up by stunted children after the age of five is limited. However, food intake should increase storage of fat and, therefore, weight.

••Physical Activity Level (PAL). Energy intake is spent in work after school or in more activity and play.

••Basal Metabolic Rate (BMR). Energy is required to maintain the healthy functioning of the body while at rest.

Catch-up growth in children and adolescent may be possible though the process is slower than catch-up in weight and it is not certain up to what age it takes place (probably up to the end of the adolescent growth spurt) [21]. All malnutrition indicators could change after the intervention (stunting, wasting and underweight), though the impact will depend on the extent of substitution effects and on whether children are increasing the use of energy for PAL and BMR. A child may have normal height and weight and still be undernourished because he does not expend enough energy in activity and play to maintain health and develop his cognitive abilities. Assessment of malnutrition should also measure PAL, particularly in adolescents who engage in considerable work and play. Unfortunately, there is no accepted theory, nor evidence, on whether children adapt to nutritional stress by reducing weight or PAL. There is also uncertainty on the definition of a minimum acceptable level of PAL (an arbitrary factor of 1.5 of BMR is often used by FAO, for example). Finally, there is no standardised way to measure PAL. Observation of behaviour in class and questionnaires for parents and teachers could be used to measure PAL.

Finally, highly deprived children may use additional energy intake from school meals simply to restore the original BMR. In addition, higher weight requires more energy; therefore, BMR is a function of body weight and the BMR requirement increases as weight increases.

Because of the complex pathways described in this section, we should not expect a strong impact of the programme on the nutritional status of children. However, we might expect an improvement in children’s activity and play and an improvement in nutritional status of siblings (if substitution effects are strong).

b. Diet diversification, micronutrients and cognition

Micronutrients may have a direct impact on cognitive abilities. It is not well understood how iron affects brain functioning and the central nervous system, but there is ample evidence that reduction in iron deficiency improves mental functions across all age groups [22,23]. Iron interventions were found to have a positive impact on infant development scales, intelligence quotient (IQ) tests and school achievement.

c. Indirect effects of better nutrition on cognition

Restoration of micronutrient requirements and energy intake can also have an impact on attention and motivation. Energy intake [24] and iron intake [22] can have an impact on hyperactivity, withdrawal, nervousness, hostile behaviour and happiness. The emotional status of children affects the attention span and has other spill-over effects. The quality of teaching in class is likely to be affected as teacher may become more motivated and as the quality of students performance in class improves (think of the different impact on learning of improving attention of 10%, 50% or 100% of students in class).

### Role of social accountability

**Figure 12:**
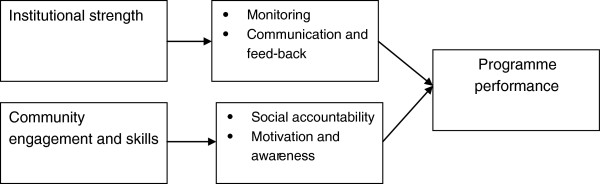
Impact of institutional and community strengthening on programme performance.

The effectiveness of the programme could be considerably improved by improving the communication mechanisms between the actors involved, by strengthening the monitoring system, and by introducing elements of social accountability. On the institutional side, the introduction of a monitoring system and the creation of communication mechanisms between the different actors would likely have the effect of improving the programme performance (see Figure 12). Similar effects could be expected from a stronger engagement of the CGSs. The delegation of certain responsibilities to the CGSs could increase motivation and awareness of the programme among beneficiaries while at the same time ensuring a better implementation of activities, (such as monitoring of public officers’ performance and food procurement from small holders in the community). These effects would have a positive impact on all the intermediate and final outcomes of the intervention.

### Main hypotheses and outcome indicators

We summarise here the expected impact of the intervention on education, nutrition and social protection discussed in Section Programme theory of the intervention.

•The intervention will have an impact on a small number of farmers in the intervention villages. Other persons in the village may benefit either directly or indirectly via an increase in income.

•The intervention will have a positive impact on enrolment, attendance and drop-out rates.

•The intervention will have an impact on cognitive abilities and class behaviour, including attention.

•The impact on learning (test scores) will be moderate as school quality is unlikely to change in the short term.

•The intervention will have a limited impact on physical growth of children because of the increase in PAL, substitution effects and the age range (6 to 17 years) of the targeted population. An impact on siblings of school-going children is possible if substitution effects are strong.

•The intervention will have a moderate impact on the diet because food purchases by communities and mayors do not follow nutritional guidelines and nutrition education is absent.

•The intervention will have little or no impact on micronutrient status as the food provision is not fortified and only moderate effects on diet diversity are expected.

•The overall impact of the programme will increase through the introduction of social accountability mechanisms and the strengthening of the monitoring and communication system.

Table 2 includes a list of the main outcome indicators of the study. Section Methods of analysis will describe how data will be collected using different survey instruments. All outcomes, including school attendance and test scores, will be obtained at the household level.

**Table 2:** Main outcome indicators of the intervention

** Indicator**	** Metric**
Income	Farm profits
Distributional effects	Small farmers participating in the programme
Schooling	Enrolment, attendance, and completion
Attention	Digit span or other test
Learning achievements	Scores on language and math tests
Physical growth	Anthropometric measures of height and weight
Diet diversity	Household consumption
Social accountability	Parental monitoring and motivation

Note that in addition to outcome indicators we will also observe the programme impact on intermediate indicators, particularly for those outcomes that are more difficult to observe directly: income and social accountability. In the case of income, we will look at intermediate outcome, such as input use (labour, land, seeds and fertiliser), investments (farm capital such as tools and machinery), and market access (marketed surplus, prices and markets). In the case of social accountability, we will observe the impact of the programme on knowledge and practices of mayors and CGSs members as they result from the training activities. The quantity, quality and timely preparation and delivery of food in school will also be examined.

## Design of the randomized evaluation

The national school feeding programme will be expanded in the regions of Mopti, Koulikoro and Kayes. These areas are among the most vulnerable of the country and offer a diversity of agro-climatic conditions and cropping patterns. Areas that are inaccessible for most of the year or in which there are serious security concerns were excluded from the study. The Ministry of Education has set clear criteria for the selection of the intervention areas. Priority is given to areas of food insecurity and vulnerability, poor enrolment rates (particularly of girls), poor presence of donors and of high community involvement. Similarly, criteria for the selection of schools include: schools with poor retention and completion rates (particularly of girls); schools with children from nomad and destitute families; schools with children with special needs; schools where one-third of class has to travel at least 3 km to reach the school; schools demanding canteens.

The impact evaluation is an integral component of the monitoring and evaluation activities of the national school feeding programme. The baseline survey is planned in intervention and control sites in 2012 and a follow-up is planned in 2014. By 2015 the control schools and community will be fully integrated in the intervention. We will consider the possibility of conducting further surveys in the following years building matched control groups in order to detect long term effects of the intervention on farmers’ productivity.

We discuss here two main elements of the evaluation approach: random assignment and manipulation of treatment; and threats to validity.

### Random assignment and manipulation of treatments

The evaluation will measure outcomes at the child, household and school level. Households and schools will be randomly assigned to the intervention. Three treatment arms are envisaged:

1) Control group. These are schools and households from villages where the intervention will not be implemented. The intervention will be delayed by at least two years in these villages, preferably without informing schools and households.

2) Regular school feeding programme group. These are schools and villages where the standard Government programme is implemented, with mayors responsible for the food procurement.

3) Home grown school feeding and social accountability group (HGSF±). These are schools and villages where the programme is implemented in addition to a capacity building component, including training of community-based organisations and local government on food procurement, nutrition education and feedback monitoring.

Note that in the intervention areas, as per the selection protocol, one school would be linked to one village. Furthermore, two schools in two villages would be selected in each commune. The HGSF+ intervention is conducted at the commune level. Training and monitoring systems involve mayors and exert their effects at the commune level, affecting outcomes in schools where the HGSF+ programme is not implemented. On the other hand, the number of communes where the programme is implemented is rather small, which reduces the statistical power of the analysis, and the effect of the school feeding intervention against the control group are best observed at the school level. Hence, we opted for a design that compares outcomes of school feeding versus a control group at the school level, and that compares outcomes of HGSF+ versus regular school feeding at the commune level. The MOE selected 58 out of the 170 most vulnerable communes in the country where the programme will be implemented. In each of these communes, mayors then selected two schools with similar characteristics that are eligible for the programme. Each school was then randomly assigned either to the treatment or to the school feeding intervention. A protocol was designed in order to ensure that contamination between the two schools in each commune was avoided. This will allow comparison of outcomes of the school feeding intervention against the control group at the school level in 58 communes. A second level randomisation involved randomly assigning the intervention schools in the 58 communes to either the regular school feeding or HGSF+ (or school feeding *plus* the enhanced training package) treatment groups. In practice, the randomisation of the HGSF+ intervention occurred using restricted randomisation at the commune level [25]. To ensure balance in the comparison groups, the random allocation of communes to school feeding or HGSF+ was undertaken by modelling pilot selection using a set of community and district level variables, testing 1,000 random allocations and selecting the permutation that minimises the R^2^ for the predicted selection [26].

Power calculations (see Appendix 1 for more details) and resource availability suggested the adoption of a sample of 25 households from the village areas of the 58 schools receiving the intervention and of 20 households in the village areas of the 58 control schools. Farmers will be oversampled in both areas in the following way: 10 out of the 25 households in the 58 intervention villages will be farmer households and 5 out of the 20 households in the 58 control villages will be farmer households. This distribution of the sample between farmers and non-farmer households and between project control groups allows the construction of comparable samples (see Table 3).

**Table 3:** Summary of sample sizes

	**Communes**	**Schools**	**Households**	**Farmers**	**Children**^**1**^
Control	58	58	870	290	2,700
HGSF	29	29	435	290	1,500
HGSF+	29	29	435	290	1,500
TOTAL	58	116	1,740	870	5,700

Note: 1) the number of children in an estimate based on an average of 2 children per family in families with children and 1.5 children per family in farmer households.

Households were randomly selected in the catchment areas of the selected schools for the survey interviews.^6^ The definition of ‘household’ in Mali is not straightforward as many families share the same living compound and polygamous families are common. A recent study [27] found that adding descriptions to a “restricted” definition of household (a group of people who normally live in your household – adopted by the DHS) increases the number of adult male members reported during the interview. This, however, does have a very limited impact on household characteristics, such as per capita food consumption, assets and agricultural production. We recommend the use of the restricted definition adopted by the Rapid Household Survey 2006, which reflects the LSMS experience in collecting data in developing countries. According to this definition, a (restricted) household is a group of people who normally live and eat together. Members spending less than three months with the household within a year should be excluded. Families living in the same compound should be considered as different (restricted) households, though the consumption section of the questionnaire was carefully designed in order to take into account the common use of food resources. Polygamous households were treated as distinct households if wives live and cook in separate houses.

In practice, the sampling of households was not conducted via a previous census as this was considered too costly in terms of time and resources. We opted for interviewing village chiefs and building a list of enlarged households in the villages covered by the sample school. The listing also included an approximation of the size of the enlarged households. In addition, a number of farmer households was oversampled in each village. Chiefs (and members of the CGSs) were asked to list which farmers they would contact if they were to purchase food within the village for the provision of school meals. This latter information was used to single out the surplus farmers in the area (up to 10 in the project villages and up to 5 in the control villages that will be interviewed).

Enlarged households were randomly selected (with inclusion probability proportional to size) from the list provided by the village chiefs, and listings of the restricted households within each selected enlarged households will then be developed through interviews with household heads. A restricted household with children aged 5 to 15 was then randomly selected within each selected enlarged non-farmer household, and we estimate that each household will have at least two children in this age group. A similar selection procedure was used for farmer households when these exceed the numbers of 10 and 5 in the project and control villages, respectively. In farmer households though, no age criteria were used and the household was identified around the main agricultural holding unit of the enlarged household. This consists of the family members involved in the main production unit (land and livestock) in the enlarged household.

### Threats to validity

The main potential threats to the internal validity of the study, including contamination, spill-over effects and Hawthorne-like effects were examined for each of the outcome indicators of Section Programme theory of the intervention. From Table 4 it seems that most threats could be avoided by:

i. assigning treatments to communes rather than to villages within communes in order to avoid contamination effects;

ii. avoid informing teachers and households of the control villages that the programme will be implemented after two years in order to avoid expectancy effects;

iii. adopting strategies in conducting cognitive and achievement tests that prevent teachers and children from over-performing.

**Table 4:** Threats to internal validity

**Indicator**	**Metric**	**Spill-over and contamination**	**Hawthorne and placebo effects**
Schooling	Enrolment, attendance, drop-out and completion	Children may attend school from neighbouring communities to have access to meals	Expectation of coming programme in control villages
Cognitive ability	Raven’s matrices or other test	Very unlikely	Teachers’ and children’s attempts to over-perform in both project and control villages
Attention	Digit span or other test	Very unlikely	Teachers’ and children’s attempts to over-perform in both project and control villages
Learning achievement	Scores on language and math tests	Very unlikely	Teachers’ and children’s attempts to over-perform in both project and control villages
Physical growth	Anthropometric measures of height and weight	Children from other villages may access school meals	Very unlikely
PAL	Parents perceptions	Very unlikely	Very unlikely
Diet diversity	Household consumption	Very unlikely	Very unlikely
Micronutrient intake	Iron status, anaemia	Children from other villages may access school meals	Very unlikely
Income	Farm profits	Unlikely, if food purchases are made in control villages	Very unlikely
Social accountability	Parental monitoring and motivation	None at household level, possible at local Government level	Possible

Given the panel structure of the data there is a potential risk of differential attrition. However, it is difficult to predict why households or farmers from the control groups should respond to the interviews in different ways. Refusal to take part in the interview by households not benefiting from the project seems to be the main threat. However, as shown in the table above, the project has limited impact on household expectations in both project and control groups and, therefore, should have limited impact on response rates.

One issue with impact on cognitive development is that the observed impact can be the result of:

•Increased attention in school resulting from the energy meal (short term effect)

•Increased overall cognitive abilities resulting from protracted school feeding, school attendance, play time, social interactions …and so on… (long term effect)

A recent systematic review of school feeding interventions [26] observed that most evaluations have not been able to distinguish the two effects. This could be achieved through careful design of the intervention as the one shown in Table 5. Half of the project children are not given the meal on the day of the test until after the test, while half of the control children are given the meal before the test. The differences across columns (a-c and b-d) should produce the long term effect, while the differences across rows (a-b and c-d) should produce the short term effect. This approach will possibly be tested as a case study in a sub-sample of schools.

**Table 5:** Teasing out short-term and long-term impact on cognitive development

	**Meal**	**No meal**
Project	a	b
Control	c	d

### Survey instruments

The impact evaluation will include household, school, farmer, village and commune level data collection.

Household questionnaire: This will collect data at the household level and for each household member separately.

The household questionnaire will include the following modules:

◦ Household roster (main demographic characteristics, including those of children residing elsewhere)

◦ Education (school attendance, education of all household members, time spent in class and working, distance and transport to school, meals while in school, parents’ aspirations, PTA membership and involvement)

◦ Household assets and farm assets (household facilities and durables, including land and livestock holdings)

◦ Economic activities (simple income questionnaire on time spent working by household members in wage work, own business and own farm)

◦ Expenditure (monetary expenditure and own production of food, education, health, durables and non-food expenditure)

◦ Anthropometry (height and weight of parents and children above six months of age – parents’ measurements are taken to assess the genetic potential)

◦ Cognitive and achievement tests (test scores on math, language and digit span test),

◦ Farm income (agricultural production and revenues, input expenditure and depreciation of farm assets)

◦ Other income (a simplified income questionnaire for other income sources like microenterprises, transfers, remittances, gifts and so on)

School questionnaire: In each school a questionnaire will be administered to head teachers and teachers, and will include the following modules:

◦ School facilities (school characteristics including boards, toilets, furniture, books and all school-feeding related characteristics – kitchen, storage room and so on)

◦ School participation (school-level data on enrolment, attendance and drop-out)

◦ School management and food procurement

◦ Teachers (qualifications, living conditions and aspirations)

◦ Training and monitoring activities

Mayors’ questionnaires: Mayors will be interviewed in each of the 58 communes at the baseline and at the endline. The purpose of the questionnaire is two-fold. First, it will collect information at the village level that will be used in the multivariate analysis when analysing the project outcomes at the household level. Second, some project outcomes, for example, the number of small farmers involved in the project, will be observed through this instrument. The tool will include the following modules:

◦ Funding of school canteens (instalments received and payments made)

◦ Food deliveries (quantities and characteristics of delivery to each school)

◦ Food procurement (details of all procurement over the calendar year)

◦ Monitoring and supervision (supervision of correct procurement and deliveries)

◦ Knowledge and practices (training and knowledge acquired)

An operations manual, including guidelines for the survey data collection was developed as part of the baseline survey training materials. The manual included background information on the survey activities, module by module guidance on the different survey tools, as well as specific guidance on the anthropometry measurements.

### Other relevant research

Complementary qualitative research will be conducted in three areas: tracking expenditure survey; parental perceptions of schooling; and assessment of programme characteristics that cannot be observed through standard surveys.

#### Public expenditure tracking

As the national programme is relatively new and in the process of scaling-up, there might be inefficiencies or leakages in the flow of funds running from the Ministry of Finance down to the school management committees. We suggest conducting research following the flow of funding from its initial allocation at the central level, to the regional offices, the mayors, the CGSs and the traders or small farmers involved. The research will allow a careful examination of all the stages of the financial transactions involved, highlighting the characteristics of the procurement system and identifying entry points for improved efficiency.

#### Focus groups with farmers, parents, children, teachers and local authority stakeholders

These focus groups are designed to obtain insights on the challenges and opportunities in terms of engaging small holder farmers in the food procurement process, determinants of parents’ and children’s decisions to attend school, as well as related issues linked to hunger and seasonality. Teacher attitudes and motivation will also be assessed. The focus groups will be conducted in a small sub-sample of communes/schools at baseline, at mid-term and during the follow-up survey.

#### Programme monitoring and process analysis

Survey data collection will be integrated in the regular project monitoring activities also supported by PCD that include school level monthly and quarterly data collection. Periodic visits (in some cases unannounced) will also be made to the project communities in order to observe nutritional characteristics of the meal served in school; pupils’ behaviour in class after the meals; modalities of cooking and storage; other aspects of project implementation that cannot be observed though a quantitative survey.

## Methods of analysis

The analysis will follow the intention to treat approach as protocol and as treated, using econometric and simulation analysis, for all the relevant outcomes of the intervention. Impact will be assessed for the different treatment arms using regression analysis to account for potential confounding variables, using a “difference-in-difference” estimator.

As enrolment rates in Mali are very low in rural areas (around 30%), one problem in conducting this analysis is that school feeding may bring to school children of very poor backgrounds with poor nutritional status and cognitive abilities to start with. This problem can be overcome econometrically by controlling for such confounders during the data analysis.

A further difficulty of difference-in-difference analysis is serial correlation [28] resulting from unobserved factors affecting the outcomes that are themselves correlated over time and that produce auto-correlated errors and invalid standard errors. Serial correlation affects estimated standard errors and can lead to erroneous acceptance or rejection of null hypotheses but not the estimation of the effect size of the intervention. Thus, it may lead to erroneously finding or not finding a statistically significant impact of the intervention. See [29] for an illustration of how this problem can be addressed by calculating clustered standard errors, a procedure that is easily implemented using the Stata software. Clustered standard errors will also be employed in all cases in which correlated outcomes are observed within the same unit of analysis, for example, when the impact of the intervention is analysed at the school level and test scores within the school are obviously correlated. Similarly, clustered standard error will be used at the household level when the project is affecting more than one child within the same family as in the case of the impact on siblings that was discussed in Section Impact on nutrition.

### Markets

We are not aware of studies of market integration in Mali. The Observatoire du Marche Agricole (OMA) collects agricultural prices on a weekly basis from more than 50 locations around the country. In principle, their data could be used to assess the extent of market integration, but not having access to the data we will assume in the following that markets are not fully integrated. If markets of staple foods (millet, sorghum and rice) are not fully integrated, prices can vary from one location to the other and the additional demand introduced by the project may have a positive impact on prices.

There are several markets of producer and consumer prices along the supply chain of staple foods. These markets are linked by complex relations and involve several actors: producers, collectors, bulkers, traders, retailers and consumers. The markets where the programme may have an impact are the *foire* (village, or group of villages, level market), the bulker market and the retailer markets. Foire markets in particular are markets where foods are purchased from local farmers by collectors and where local consumers make their purchases. If the project has an impact on prices it is likely to occur at this level. In practice, the impact on prices can happen a maximum of three times per year when purchases by the mayors are made and only in the HGSF+ programme sites.

Impact on prices could in principle be observed through the household level questionnaires. The farm gate price could be observed at the household level by including in the questionnaire questions related to prices paid and time of sales. This, however, would complicate the income section of the farmer questionnaire. Consumer prices are more difficult to observe in a standard household survey because the recall time is 7 or 30 days and there is only one survey per year.

As part of the programme monitoring activities, price data will be collected on a monthly basis for millet, sorghum and rice in the local foire next to each of the selected schools. The work could be assisted by the OMA through the provision of training given its long experience in the field. Collection of prices does not even require visits to markets if stable contacts can be established with collectors in each of the markets and prices could be communicated by phone.

### Other general equilibrium effects

There is a possibility that some of the outcomes of the programme cannot be observed because of the type of intervention and the sampling design. In particular, the programme is likely to benefit only a few farmers in each of the project villages and the number of project villages for the study may not be sufficient to perform project-control comparisons that are statistically significant. In addition, there is a risk of large fluctuations within the trial period, which would tend to increase the variability in the outcomes and process measures – and, hence, possibly fatally undermine the power of the study due to this increased variability. We developed two strategies to mitigate this risk. The first involved the design of the study: we worked with Ministry of Agriculture technical stakeholders to spread the intervention area across a broad range of agro-ecological zones across the country. More importantly though, we devised an analysis strategy that includes two different simulations of the potential impact: The first is a micro-simulation of the farm-level impact of the intervention. Using household data we can estimate production and profit functions and then simulate the impact on farmers’ income, factoring in the additional demand and changes in food prices. Changes in prices can be simulated or observed directly through surveys. Similarly, the impact on consumption can be simulated after the estimation of a consumption function using household data. The second exercise consists of a village level simulation using a computable general equilibrium model. In this case, data are collected in one or two villages in order to build a social accounting matrix (SAM) to be used to simulate the impact of the injection of liquidity in the village economy. The advantage of this approach is that it allows the simulation of the impact on the entire community via price and demand effects. This method could also be used to simulate the differential impact of the programme in a drought and in a surplus year.

## Heterogeneity of impact

Gender, age and geographic area are other relevant categories to analyse impact. The impacts of school feeding in different contexts are quite heterogeneous [30]. School feeding, for instance, has been associated with marked improvements in school participation of girls in rural areas with large gender disparities in access to education [31]. Small-holder farmers targeted by the program will in large proportions be women. From the educational perspective, school feeding impact has also been found to vary with pupil age, as household schooling decisions are also affected by the opportunity costs of education, that tend to change with gender and increasingage and [32].

The programme is targeted to disadvantaged groups. Main beneficiaries are:

•Children aged 6 to 17 attending primary school

•Poor, rural districts of the country

•Smallholder farmer households

The programme has a potential poverty inequality reduction impact at the national level. At the local level it has a potential poverty reduction impact, but the inequality reduction impact will depend on whether:

•The project will increase enrolment. Children going to school are likely to be from richer background and more accessible areas.

•The project will involve small farmers. The programme might rely on large farmers or traders for the provision of food.

## Cost effectiveness

Cost data will be collected retrospectively following an ingredients approach using a semi-structured questionnaire. The survey will be based on a standardised costing framework capturing capital (fixed) and recurrent costs incurred at the school level. The questionnaire will also cover both cash and in-kind contributions and will be used to estimate both financial and economic costs. Financial costs capture actual expenditures in terms of programme implementation on an annual basis. Economic costs included the opportunity costs of community members, teaching staff and other school level stakeholders involved in the school feeding service provision. Opportunity costs of the school staff and community members will be calculated using local pay scales. Capital costs will be annuitized over the useful life of all relevant school level assets using a discount rate of 3% as per World Bank recommendations. Annuitisation enables an equivalent annual cost to be estimated and reflects the value in-use of capital items, rather than reflecting when the item was purchased [33].

Process and output data covering the adequacy of the service delivery will be collected from monitoring visits on a quarterly basis using standardised data collection forms. Output data will be combined with the costs to provide estimates of cost-efficiency metrics, including costs per beneficiary, kilocalories, iron and vitamin A delivered. Sensitivity analysis will be undertaken to account for uncertainties in the economic evaluation. The figures obtained in this way will then be compared to figures calculated for other interventions.

Of particular interest is the cost-effectiveness of the community level/social accountability component of the intervention. The comparison between the HGSF+ and the regular GSFP is roughly equivalent to the comparison between a home grown school feeding project and a standard school feeding project. Many would expect HGSF to be cheaper and more cost effective because of lower transport costs. However, the alternative procurement source, its distance and affordability is unknown, and hence the difference in costs between the two programmes is an empirical question.

## Risks

Overall, the study involves very low risks for participants. The risks are related to the quality of the food service delivered by the national school feeding programme. Risks involve food hygiene, sanitation and preparation related issues. There is also a risk associated with the potential disruption in the service delivery that could undermine the potential impact. If parents believe a child is fed in school when in fact the food service is not functioning, then the child can be potentially worse off after the intervention, though possibly just for one or a few days. The study will examine these risks in detail and also identify and test strategies to minimize these risks operationally.

## Ethical clearance

Ethical clearance was obtained from the appropriate boards in Mali and at Imperial College, London. Meetings were held from the early stages in the study development with relevant Government Ministries both at central and decentralised levels to discuss the purpose, procedures and risks involved in the study. Informed consent was obtained from parents/guardians of children through written and verbal information provided before interviews.

### Trial oversight

The evaluation team will be guided by a steering group, including the Government of Mali’s coordination framework on school feeding, and the Ministry of Education and Ministry of Agriculture. An independent steering group of experts in evaluation, public health, agriculture and education provided peer reviews prior to the data collection and will be convened at completion of the study to approve the final data analysis plan.

## Discussion

National Governments in sub-Saharan Africa have demonstrated strong leadership in the response to the recent food and financial crises by scaling-up school feeding programmes. “Home-grown” school feeding programmes have the potential to link the increased demand for school feeding goods and services to community-based stakeholders, including small-holder farmers and women’s groups. However, there is a dearth of evidence on the costs and benefits of school feeding programmes linked to small-holder agriculture and community development. This evaluation will be the first to examine the impact of linking school food service provision on small-holder farmer income, as well as the link between community level engagement and programme performance, alongside assessing the more traditional benefits to school children.

The impact evaluation follows a theory-based approach and is underpinned by the development of the programme theory for the intervention. It includes a process evaluation component that will enable policymakers to not only understand if the intervention works or not, but also why and how it is working. By using a mixed-method approach, the evaluation will explore both hard outcomes (for example, enrolment, income, welfare) but also perceptions on softer elements of programme performance (for example, community ownership and accountability).

This study will also be the first evaluation designed around the scale-up of a national school feeding programme in sub-Saharan Africa. It is a prime opportunity to provide policy relevant evidence. To ensure that this was the case, policy makers across Education, Health, Agriculture and Local Government Ministries were engaged from the early design stages through to survey design and implementation. In particular, this study is anchored in the framework provided by the Ministry of Education’s recently developed national school feeding policy. In Mali, the national policy on decentralisation also meant that policy makers at regional and commune level be engaged in design process. A number of meetings were held to sensitise stakeholders at the different levels, also including them in the randomisation process.

Conducting this type of research in the Malian context also raises a number of practical issues, including logistics, security and local political constraints. A set of contingency plans were developed to tackle foreseen challenges; however, the major upheaval currently taking place in the country clearly poses a major challenge to the intervention itself. The scale-up areas, however, remain under Government control, and recent news provides encouraging signs that the work will continue. Despite these challenges, the study is underway, and the baseline survey will provide some important new data on the links between education, health and small-holder agriculture in food insecure areas of Mali.

## Trial status

As of April 2012, the baseline survey for this study is underway though completion is pending, depending on the current political unrest in the country.

## Appendix 1: Power calculations

The designed adopted by this study consists of (a) a multi-site cluster randomized trial to detect the impact of the regular school feeding and HGSF+ intervention against a control group without school feeding intervention, and of (b) a cluster randomized trial to detect the impact of the HGSF+ intervention against the regular school feeding intervention.

### Comparing school feeding to no-school feeding (multi-site cluster randomized trial)

In the multi-site cluster randomized trial the sites are blocks and clusters are randomly assigned to treatment and control within each site. In each commune (site), two schools (clusters) are selected and randomly assigned to the treatment and the control groups. This is equivalent to conducting a randomization of schools stratifying by commune.

There are a total of 58 communes selected for the intervention and two schools are chosen by local education authorities in each commune. In each school/village, 25 households will be interviewed. Only families with children in the age range from 5 to 15 will be selected, which implies that some 55 children in each community and a total of more than 6,500 children will take part to the interviews. The average number of children aged 5 to 15 in rural Mali was 2.58 in 2006 based on the data collected by the DHS. Care will also be taken to obtain a sizable sample of farmers and potential providers of staples for school feeding in each village.

We calculate power and minimum detectable effect size for the following outcomes:

•enrolment rates

•test scores

•farm income

### Enrolment

Public education in Mali is compulsory for nine years from age 7 to age 15 and split in a six-year primary cycle and a three-year lower secondary cycle. Enrolment rates in rural areas are very low and lower for girls compared to boys. Table 6 below reports the attendance rates for the age groups 7 to 12 and 13 to 15 in rural Mali calculated from the DHS survey of 2006 (Table 6).

**Table 6:** Attendance rates for the 7 to 12 and 13 to 15 age groups in rural Mali (calculated from DHS, 2006)

	**Primary (age group 7 to 12)**	**Lower secondary (age group 13 to 15)**	**Age group 7 to 15**
All	35.6	33.8	35.1
Male	39.1	39.9	39.3
Female	32.1	27.4	30.8

Ahmed [34] found a 14% difference in enrolment rates between project and control groups in Bangladesh. Researchers for FAO/WHO [21] found an effect of 6% in Burkina Faso, Jacoby [35] found a difference of 3% in Peru, while Buttenheim *et al*. [36] found no effect in Laos, mostly due to lack of intervention implementation. We adopt an increase in attendance rate by five percentage points as a minimum expected impact of the programme. Simulations of power for the percent difference in enrolment in the project group versus the control group show that a 5% difference will be detected with 80% probability, while differences of 10% and 15% will be detected with certainty. At 80% power, the minimum detectable difference is 5%.

### Test scores

Few studies have investigated the impact of school feeding on learning outcomes. Drèze *et al*. [32] reviewed the experimental evidence on the impact of school feeding on standardised test scores and found an average standardised effect size of 0.31 for math achievement tests, while no effect was found on reading tests. We adopt a 0.3 difference as the expected effect of the intervention on math test scores. Based on the Scrochet report, the recommended intra-cluster correlation coefficient for math and reading tests is 0.15. We also assume that only about half of the children interviewed will be able to take the test and we set the number of children tested per village at 25. Simulations of power as a function of minimum standardised detectable effect show that a standardised difference of 0.1 would be detected in 24% of cases, a difference of 0.2 would be detected in 68% of cases and a difference of 0.3 would be detected in 95% of cases. With power set at 80%, the minimum detectable difference is 0.23.

### Income

No previous studies have investigated the impact of home-grown school feeding on farmers’ incomes and indeed this type of programme is entirely new. In addition, it is difficult to find programmes with effects similar to those of school feeding programmes. The Purchase for Progress programme by the World Food Programme, which similarly to the Malian school feeding programme purchases food for local producers, has modelled an increase in income by $50 dollars as a reasonable target for small African farmers. With a per capita GDP of $700 this corresponds to 7.5% of the average Malian income, though the income of small farmers is likely to be less than half the national average income. In addition, farm income is about 50% of total household income and the impact of a $50 increase on farm income is therefore much larger. We adopt 15% and 30% as minimum detectable effects of the programme on agricultural incomes of small farmers.

We adopt a conservative estimate of 10 farmers surveyed in each village, and based on data on agricultural incomes of Ghanaian farmers, we estimated the intra-class correlation coefficient (ϱ = 0.25) and the variance explained by site variability (B = 0.40). Note that given the high value of the standard deviation of income, similar in size to average income, the standardised difference and the percentage difference are almost equivalent. The probability of detecting an increase by 15% in agricultural income is 35% and the probability of detecting an impact of 30% is 88%. A power of 80% will be able to detect an income increase by 26%.

### Comparing school feeding to HGSF+ (cluster randomized trial)

To estimate MDES we assume that 58 clusters (schools) will be randomly assigned to regular school feeding or HGSF+. Using standard simulations we observe that

•A 5% difference will be detected with 60% probability, while differences of 10% and 15% will be detected with certainty. At 80% power the minimum detectable difference is 6%.

•A standardised difference of 0.1 would be detected in 15% of cases, a difference of 0.2 would be detected in 41% of cases and a difference of 0.3 would be detected in 79% of cases. With power set at 80% the minimum detectable difference is 0.32.

•The probability of detecting an increase by 15% in agricultural income is 17% and the probability of detecting an impact of 30% is 50%. A power of 80% will be able to detect an income increase by 40%.

### Conclusions

The power analysis suggests that the study will be able to detect an increase in enrolment by 5%, a 0.2 difference in test scores and an increase in farmers’ incomes by 25% between the school feeding interventions and the control group. The study will be able to detect changes in enrolment by 6%, differences in tests scores by 0.3 and changes in income by 40% between the regular school feeding and the HGSF+ components of the intervention see (Table 7).

**Table 7:** Summary table of values used in the power calculations

	**Enrolment**	**Test scores**	**Agricultural income**
Alpha	0.05	0.05	0.05
N (observations)	55	25	10
J (schools)	2	2	2
K (clusters)	58	58	58
Intra Cluster Correlation	-	0.15	0.25
Between sites variability	-	0.20	0.40

While the sample size is well suited to the analysis of programme impact on attendance rates and test scores, it is clearly insufficient for the analysis of income effects of the HGSF+ intervention. However, we hope to overcome this problem in two ways: by increasing statistical power through restricted randomisation and by focussing on intermediate outcomes rather than on household income, a variable that has large variance and high within cluster correlation. First, in order to increase statistical power we will adopt ‘restricted randomisation’ [25]. We will use the baseline data collected at the village and school levels to remove the randomisation outcomes that would result in an unbalanced comparison of project and control communes. The randomisation of the HGSF+ component of the project within the 58 communes will be performed only within the restricted sample of balanced randomisation outcomes. Second, we will analyse intermediate indicators of household income such as input use (labour and other farm inputs, such as land, seeds and fertiliser) and farm capital use like animal traction, tools and simple machinery.

## Endnotes

^1^ Source: UNESCO Institute of Statistics Mali country profile, 2009.

^2^ Source: Mali Demographic and Health Survey, 2006.

^3^ Ibid, MDHS, 2006.

^4^ Notice that there is a potential nutritional downside on nutrition following from this. If parents believe a child is fed in school when he/she is not, then the child is worse off after the intervention, though possibly just for one or few days.

^5^ Note that in the econometric model we assumed that the schooling choice was made entirely by the parents ignoring the emotional effect of school feeding on children. School fed children may become more motivated and this could lead to higher attendance directly or indirectly (via parents’ decisions). This suggests that the model should include the utility function of children, not just the parents [15].

^6^ We suggest a random selection of households in the villages. This approach has also been successfully adopted, for example, by [22,27]. One additional advantage of this approach is that it allows the identification of the determinants of enrolment/attendance and provide estimates of the relative relevance of school cost reduction effects produced by the programme.

## Abbreviations

BMR: Basal Metabolic Rate; CAADP: Comprehensive Africa Agriculture Development Programme; CAP: Centres d’ Animation Pedagogiques; CFA: Communauté Financière Africaine; CGS: Comité de *Gestion Scolaire*; CRS: Catholic Relief Services; DHS: Demographic and Health Surveys; FAO: Food and Agricultural Organization of the United Nations; GDP: Gross Domestic Product; GSFP: Government School Feeding Programme; IDS: Institute of Development Studies; IPA: Innovations for Poverty Action;IQ: Intelligence Qotient; HGSF: Home-Grown School Feeding; J-PAL: Abdul Latif Jameel Poverty Action Lab; LIFDC: Low Income Food Deficit Country; LSMS: Living Standards Measurement Surveys; M&E: Monitoring and Evaluation; MDES: Minimum Detectable Effect Size; MDHS: Mali Demographic Health Survey; MOE: Ministry of Eduacation; NEPAD: New Partnership for Africa’s Development; OMA: Observatoire du Marche Agricole; P4P: Purchase for Progress; PAL: Physical Activity Level; PCD: Partnership for Child Development; PPP: Purchasing Power Parity; PTA: Parents Teachers Association; RCT: Randomised Controlled Trial; SAM: Social Accounting Matrix; SNV: Stichting Nederlandse Vrijwilligers (Foundation of Netherlands Volunteers); UNDP: United Nations Development Programme; UNESCO: United Nations Educational, Scientific and Cultural Organization; USD: United States Dollars; USDA: United States Department of Agriculture; WFP: World Food Program; WHO: World Health Organisation

## Competing interests

The authors declare that they have no competing interests.

## Authors’ contributions

EM led the development of the agriculture dimension whilst AG led the development of the school feeding dimension of the paper. Both authors read and approved the final manuscript.

## References

[B1] Bundy DAP Burbano C Grosh M Gelli A Jukes M Drake L Rethinking School Feeding: Social Safety Nets, Child Development, and the Education Sector 2009 Washington, DC: World Bank

[B2] Sumberg J Sabates-Wheeler R Linking agricultural development to school feeding in Sub-Saharan Africa: theoretical perspectives Food Policy 2011 36 341 349 10.1016/j.foodpol.2011.03.001

[B3] New Partnership for Africa’s Development The NEPAD Home-Grown School Feeding Programme: A Concept Note 2003 Addis Ababa: NEPAD

[B4] Gelli A Neeser K Drake L Home Grown School Feeding: Linking Small Holder Agriculture to School Food Provision PCD Working Paper No. 212, Volume 2010 London: Partnership for Child Development

[B5] World Food Programme Comprehensive Food Security and Vulnerability Assessment 2005 Bamako: World Food Programme

[B6] USDA Foreign Agricultural Service Assessment of Local Production for School Feeding in Mali 2009 Washington, DC: United States Department of Agriculture

[B7] Partnership for Child Development Anaemia in school children in eight countries in Africa and Asia Public Health Nutr 2001 4 749 756 11415481 10.1079/phn2000111

[B8] World Food Programme/Partnership for Child Development/World Bank School feeding in Mali Situation Analysis 2011 Bamako: World Food Programme

[B9] Johnson C Janoch E Engaging Communities: Evaluating Social Accountability in School Feeding Programmes 2011 London: Partnership for Child Development

[B10] Ahmed AU Sharma M Home Grown School Feeding Project: Economic Modeling and Supply Response 2007 Washington DC: IFPRI

[B11] Baulch B Hansen H Trung LD Minh Tam TN The spatial integration of paddy markets in Vietnam J Agric Econ 2008 59 271 295 10.1111/j.1477-9552.2007.00148.x

[B12] Glewwe P Schools and skills in developing countries: education policies and socioeconomic outcomes J Econ Lit 2002 40 436 482 10.1257/002205102320161258

[B13] Jukes MCH Drake LJ Bundy DAP School Health, Nutrition and Education for All: Levelling the Playing Field 2008 Oxfordshire, UK: CAB International

[B14] Galloway R Kristjansson EA Gelli A Meir U Espejo F Bundy DA School feeding, cost and outcomes Food Nutr Bull 2009 30 171 182 19689096 10.1177/156482650903000209

[B15] Ahmed AU Arends-Kuenning M Do crowded classrooms crowd out learning? Evidence from the Food for Education programme in Bangladesh Food Consumption and Nutrition Division Discussion Paper 149, Volume 2003 Washington, DC: International Food Policy Research Institute 10.1177/156482650302400409 14870628

[B16] Kristjansson E Francis DK Liberato S Benkhalti Jandu M Welch V Batal M Greenhalgh T Rader T Noonan E Shea B Janzen L Wells GA Petticrew M Feeding interventions for improving the physical and psychosocial health of disadvantaged children aged three months to five years (Protocol) Cochrane Database of Systematic Reviews 2012 6 Art. No. CD009924 10.1002/14651858.CD009924 PMC6885042 25739460

[B17] Beaton GH Ghassemi H Supplementary feeding programs for young children in developing countries Am J Clin Nutr 1982 35 863 916 7039297 10.1093/ajcn/35.4.864

[B18] Svedberg P Poverty and Undernutrition: Theory, Measurement and Policy 2000 Oxford: Oxford University Press

[B19] Kazianga H de Walque D Alderman H Educational and Health Impacts of Two School Feeding Schemes: Evidence from a Randomized Trial in Rural Burkina Faso World Bank Policy Research Working Paper 4976, Volume 2009

[B20] Adelman S Alderman H Gilligan DO Lehrer K The Impact of Alternative Food for Education Programs on Learning Achievement and Cognitive Development in Northern Uganda 2008 Washington, DC: World Bank

[B21] FAO/WHO Energy and Protein Requirements Technical Report Series 724, Volume 1985 Geneva: WHO 3937340

[B22] Grantham-McGregor SG Ani G Iron-deficiency anemia: reexamining the nature and magnitude of the public health problem J Nutr 2001 131 649S 668S 11160600 10.1093/jn/131.2.697S

[B23] Pollitt E Iron deficiency and cognitive function Annu Rev Nutr 1993 13 521 537 10.1146/annurev.nu.13.070193.002513 8369157

[B24] Polliltt E Gersowitz M Gargiulo M Educational benefits of the United States school feeding program: a critical review of the literature Am J Public Health 1978 68 477 481 10.2105/AJPH.68.5.477 347957 PMC1653885

[B25] Hayes R Moulton LH Cluster Randomised Trials 2009 Oxford, UK: Chapman & Hall/CRC Press

[B26] Bruhn M McKenzie D In Pursuit of Balance: Randomization in Practice in Development Field Experiments World Bank Policy Research Working Paper 4572, Volume 2009 Washington, DC: World Bank

[B27] Beaman L Dillon A Do household definitions matter in survey design? results from a randomizes survey experiment in Mali J Dev Econ 2012 98 124 135 10.1016/j.jdeveco.2011.06.005

[B28] Angrist JD Pischke JS Mostly Harmless Econometrics 2009 Princeton, NJ: Princeton University Press

[B29] Bertrand M Duflo E Mullainathan S How much should we trust differences-in-differences estimates? Q J Econ 2004 119 249 275 10.1162/003355304772839588

[B30] Kristjansson EA Robinson V Petticrew M MacDonald B Krasevec J Janzen L Greenhalgh T Wells G MacGowan J Farmer A Shea BJ Mayhew A Tugwell P School feeding for improving the physical and psychosocial health of disadvantaged elementary school children Cochrane Database Syst Rev 2007 CD004676 17253518 10.1002/14651858.CD004676.pub2

[B31] Gelli A Meir U Espejo F Does provision of food in school increase girls’ enrollment? Evidence from schools in Sub-Saharan Africa Food Nutr Bull 2007 28 149 155 10.1177/156482650702800203 24683673

[B32] Drèze J Kingdon G School participation in rural India Rev Dev Econ 2001 5 1 24 10.1111/1467-9361.00103

[B33] Brooker S Kabatereine NB Fleming F Devlin N Cost and cost-effectiveness of nationwide school-based helminth control in Uganda: intra-country variation and effects of scaling-up Health Policy Plan 2008 23 24 35 18024966 10.1093/heapol/czm041 PMC2637386

[B34] Ahmed AU Impact of Feeding Children in School: Evidence from Bangladesh 2004 Washington, DC: IFPRI

[B35] Jacoby HG Is there an intrahousehold 'flypaper' effect? Evidence from a school feeding programme Econ J 2001 112 196 221

[B36] Buttenheim A Alderman H Friedman J Impact Evaluation of School Feeding Programs in Lao PDR World Bank Policy Research Working Paper 5518, Volume 2010

